# Supplementary material for: Syphilis and Co-Infections with HIV-1, HBV, and HCV among People Who Use Crack-Cocaine in Northern Brazil
Source: Pathogens. 2022 Sep 16;11(9):1055. doi: 10.3390/pathogens11091055 (PMC9502650; doi:10.3390/pathogens11091055)
Supplement: Supplementary file 1 [file pathogens-11-01055-s001.zip › pathogens-1859677-supplementary.pdf]

SUPPLEMENTARY MATERIAL

Full Title: Syphilis and co-infections with HIV-1, HBV and HCV among people who use crack-cocaine in northern Brazil.

Authors: Karen Lorena N. Baia, Ana Caroline C. Cordeiro, Paula Cristina R. Frade, Allana Gabrielly N. Gouveia, Rafael Lima Resque, Luiz Marcelo L. Pinheiro, Ricardo Roberto S. Fonseca, Luiz Fernando A. Machado, Luisa C. Martins, Emil Kupek, Benedikt Fischer, and Aldemir B. Oliveira-Filho.

Table S1. Sample size of people who use crack-cocaine (PWUCC) assessed in each municipality in the states of Amapá and Pará, northern Brazil.

Municipality	Number in figure 1	Brazilian state	Number of PWUCC accessed
Soure	1	Pará	14
Salvaterra	2	Pará	11
Ponta de Pedras	3	Pará	9
Curralinho	4	Pará	38
Breves	5	Pará	86
Melgaço	6	Pará	12
Belém	7	Pará	58
Benevides	8	Pará	11
Bragança	9	Pará	165
Castanhal	10	Pará	90
Marituba	11	Pará	40
Abaetetuba	12	Pará	19
Cametá	13	Pará	13
Capanema	14	Pará	113
Marabá	15	Pará	25
Parauapebas	16	Pará	18
Altamira	17	Pará	13
Santarém	18	Pará	29
Tucuruí	19	Pará	17
Augusto Correa	20	Pará	12
Laranjal do Jari	21	Amapá	22
Macapá	22	Amapá	61
Mazagão	23	Amapá	26
Santana	24	Amapá	59
Vitória do Jari	25	Amapá	15
Porto Grande	26	Amapá	14

Table S2. Venereal Disease Research Laboratory (VDRL) results and titers among people who use crack-cocaine in northern Brazil.

VDRL	N (%)
Plasma (n = 287)	
Positive	238 (82.9)
Negative	49 (17.1)
Titers (n = 238)	
1:16	43 (18.1)
1:32	88 (36.9)
1:64	54 (22.7)
1:128	28 (11.8)
1:256	18 (7.6)
1:512	7 (2.9)

Table S3. Factors not associated with syphilis among people who use crack-cocaine in northern Brazil using logistic regression models.

Factors	Total	Syphilis + (%)	Bivariate OR (95% CI)	Multivariate OR (95% CI)
Male *versus* (*vs.*) Female	720	199 (27.6)	0.8 (0.6 – 1.1)	1.2 (0.7 – 1.5)
Non-white *vs.* White	871	250 (28.7)	1.1 (0.7 – 1.7)	1.5 (0.6 – 2.3)
Not married *vs.* Married†	933	271 (29.0)	1.1 (0.5 – 1.9)	1.4 (0.6 – 2.2)
Unstable housing (including homelessness) *vs.* Stable housing†	157	52 (33.1)	1.3 (0.9 – 1.8)	1.5 (0.8 – 2.8)
Sharing of crack-cocaine use equipment *vs.* No equipment sharing	327	106 (32.4)	1.3 (0.9 – 1.7)	1.6 (0.8 – 2.6)
Heterosexual vs. Same sex	901	264 (29.3)	1.2 (0.7 – 2.0)	1.5 (0.6 – 2.4)
Oral sex *vs.* No oral sex†	602	181 (30.1)	1.1 (0.9 – 1.6)	1.4 (0.8 – 2.0)
Anal sex *vs.* No anal sex†	311	98 (31.5)	1.2 (0.8 – 1.6)	1.5 (0.8 – 2.1)
No access to public health service†	902	269 (29.8)	1.7 (0.9 – 2.8)	2.4 (0.8 – 3.4)

†Last 12 months. OR: Odds Ratio. 95% CI: 95% confidence interval.

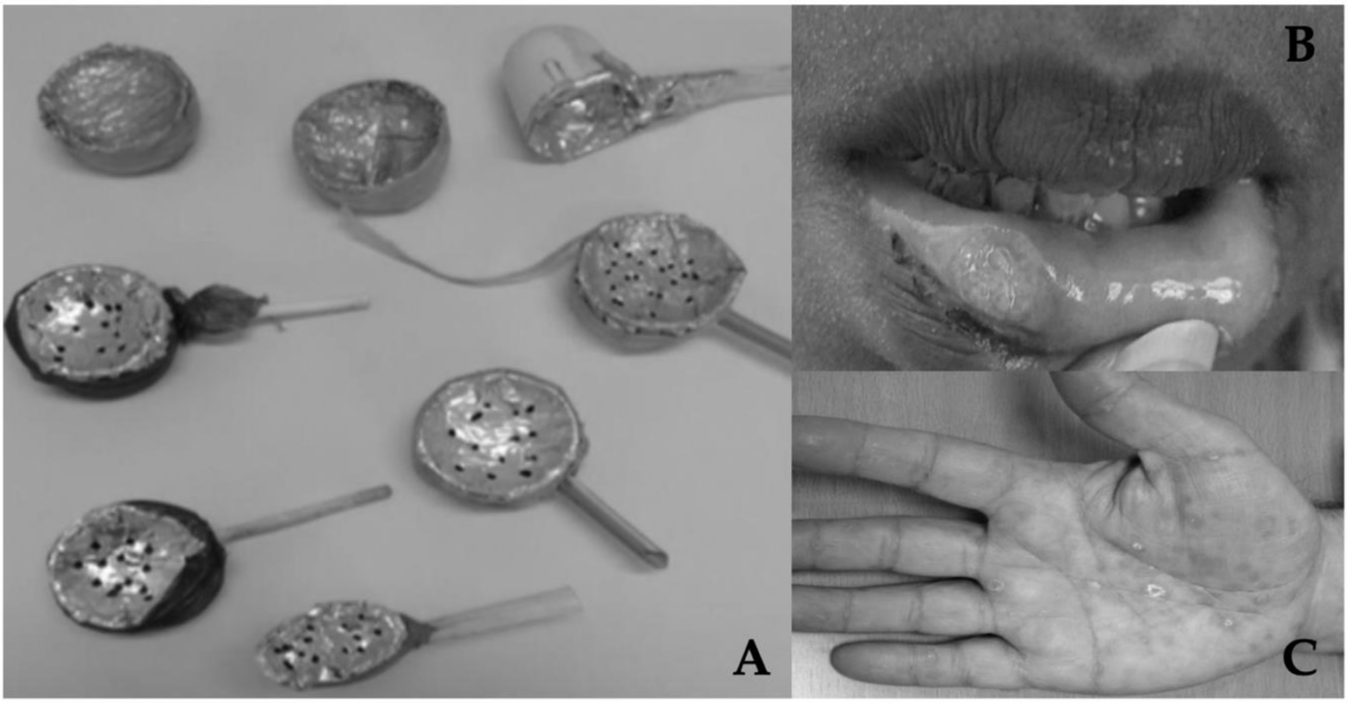

Figure S1. Characteristics detected among people who use crack-cocaine in northern Brazil. Reservoir manually made for crack-cocaine use (A). Lip lesion (B). Skin lesion (C).
